# Enhancing Knowledge and Confidence in Wilderness Medicine Through Virtual Education: Lessons From a National Boot Camp

**DOI:** 10.7759/cureus.78689

**Published:** 2025-02-07

**Authors:** Alexander T Lato, Arthur S Berg, Jessica A Parascando, Shawn F Phillips, Cayce A Onks, Jayson R Loeffert

**Affiliations:** 1 Department of Family and Community Medicine, Penn State Health St. Joseph Medical Center, Reading, USA; 2 Department of Public Health Sciences, Penn State College of Medicine, Milton S. Hershey Medical Center, Hershey, USA; 3 Department of Family Medicine, University of Colorado Anschutz Medical Campus, Aurora, USA; 4 Department of Family and Community and Orthopedics and Rehabilitation, Penn State College of Medicine, Milton S. Hershey Medical Center, Hershey, USA

**Keywords:** graduate medical education, primary care sports medicine, survey, training, wilderness medicine

## Abstract

Wilderness medicine (WM) education is an important aspect of training for sports medicine fellows, preparing healthcare providers to address medical challenges in remote and austere environments. It is traditionally taught in immersive, in-person settings. The COVID-19 pandemic prompted a rapid shift toward virtual education platforms, challenging traditional teaching methods. This study evaluates the efficacy of a virtual WM boot camp to teach WM concepts, converting an established in-person program into a national, virtual educational opportunity. Participants included Primary Care Sports Medicine fellows and attendings located in the USA, with data collected over two years (2020 and 2021). Pre- and post-surveys assessed comfort levels and knowledge before and after attending the virtual boot camp. Results indicate significant improvements in comfort and knowledge across various WM topics for both fellows and attendings. Limitations include the absence of hands-on experiences and the inability to fully replicate simulated scenarios. This study underscores the adaptability and potential of virtual education in WM and discusses the benefits of virtual over in-person education. It further demonstrates the potential for virtual training in medical education more broadly, optimizing medical training in a post-pandemic era.

## Introduction

The COVID-19 pandemic ushered in a transformative era in education. With traditional in-person learning avenues disrupted by lockdowns and travel restrictions, educators worldwide turned to virtual teaching platforms [1]. This transition provided a way to ensure continuity of education across all educational levels, including K-12, undergraduate, and medical education [2-4]. Although existing technology was immediately available to continue education efforts, there are many questions about its success. As such, we desired to understand if virtual platforms successfully meet the goals of traditional educational environments.

We sought to answer this question within the context of wilderness medicine (WM) education. WM is an important aspect of sports medicine fellowship education that prepares healthcare providers to navigate medical challenges in remote and austere environments. Traditionally delivered through immersive, in-person experiences, such as destination trips or WM conferences, WM education also faced the challenge of social distancing during the COVID-19 pandemic, leading WM education to adopt virtual education platforms. Even prior to the pandemic, WM education was difficult for many Primary Care Sports Medicine (PCSM) programs due to cost and the need to travel to remote locations often.

Our research represents an evaluation of the educational efficacy of a virtual WM boot camp - an initiative born out of the necessity to convert an already established in-person WM boot camp program into a virtual educational experience, as previously described by Loeffert et al. [5]. To address the quality and educational impact of the boot camp, our investigation focused on assessing the improvement in knowledge and comfort levels of PCSM fellows and attendings from across the USA in six key WM topics after attending the virtual boot camp. Of the topics covered in the boot camp, four are considered most crucial in WM, with the fifth ranking among the top 10 as determined by a recent publication from Mikros et al. [6]. The data analyzed included assessments of comfort in and knowledge of these WM concepts among attendees before and after boot camp participation.

The primary aim of our research was to evaluate whether a virtual WM boot camp can effectively increase knowledge and comfort levels in key WM topics among PCSM fellows and attendings. We intend to do so by demonstrating increased levels of comfort with and understanding of WM concepts among participants in a virtual WM boot camp, collecting pre- and post-boot camp surveys and assessments. The secondary aim was to examine the benefits, limitations, and broader applicability of virtual education for medical training, particularly for optimizing education delivery in a post-pandemic era. The hypothesis is that virtual education is an effective tool for teaching and learning.

## Materials and methods

Curriculum design 

The virtual WM boot camp was designed in response to the recommendation for social distancing in 2020 and the years following, secondary to the COVID-19 pandemic. Specifics of the boot camp were published previously by Loeffert et al. [5]. Topics covered in the boot camp were identified by the Accreditation Council for Graduate Medical Education (ACGME) sports medicine fellowship content recommendations and concepts covered within WM-emergency medicine fellowships [7]. The schedule for the day is presented in Tables 1-2. 

**Table 1:** 2020 Virtual Wilderness Medicine Bootcamp Agenda

Agenda reflects Eastern Standard Time		Group
10:10 AM	Conference start - introduction	
10:50	Hypothermia	A
11:30	Hyperthermia	B
12:10	Altitude sickness	C
12:10	Break	
12:20	Envenomation + lightning strike	A
1:00	Mass casualty	B
1:40	Improvisational splinting	C
2:00	Communication/extraction decision-making	B
2:20	Wilderness medicine kit	C
2:40	Wrap up	
2:50	Adjourn	

**Table 2:** 2021 Virtual Wilderness Medicine Bootcamp Agenda

Agenda reflects Eastern Standard Time		Group
9:30 AM	Conference start - introduction	
9:40	Hypothermia	A
10:20	Hyperthermia	B
11:00	Altitude sickness	C
11:40	Break	
11:50	Envenomation + lightning strike	A
12:30	Mass casualty	B
1:10	Improvisational splinting	C
1:40	Break	
1:50	Scuba and dive medicine	A
2:30	Communication/extraction decision-making	B
3:10	Wilderness medicine kit	C
3:30	Wrap up	
3:40	Adjourn	

Presenters

All presenters were faculty members of an ACGME-accredited PCSM fellowship program. Each faculty member was Certificate of Added Qualification (CAQ) certified in sports medicine and had a range of teaching experience from three to 14 years (average 7.3 years). Each presenter had prior experience delivering virtual presentations and was familiar with the virtual platform prior to the boot camp.

Participants

Boot camp participants included PCSM fellows from across the USA and their faculty members. Using the PCSM fellowship listserv, an email was generated to contact all fellowship program directors and notify them of the opportunity. This listserv was accessed through the presenting institution’s PCSM fellowship program. Every PCSM fellowship across the country was contacted, and all of their fellows and fellowship faculty were invited to attend regardless of their residency training background. Inclusion criteria were fellows or faculty involved in a PCSM fellowship program. The only exclusion criterion was that only fellows and their faculty members were invited to attend. It is difficult to know exactly how many participants took part in each virtual boot camp, given the fluidity of audience participation and engagement. Most participants, including fellows and faculty, registered and logged on to the event throughout the day, were 161 in 2020 (199 registered to attend from 65 fellowships) and 112 in 2021 (131 registered from 51 fellowships). The variety of participants’ primary specialty backgrounds is presented in Table 3. 

**Table 3:** Participant Primary Specialty Backgrounds by Year

	2020			2021		
	Total	Fellows	Attendings	Total	Fellows	Attendings
Total participants	156	102	54	123	85	38
Family medicine, n (%)	97 (62.1%)	60 (58.8%)	37 (68.5%)	85 (69.2 %)	56 (65.9%)	29 (76.3%)
Internal medicine, n (%)	9 (5.8%)	7 (6.9%)	2 (3.7%)	7 (5.7%)	3 (3.5%)	4 (10.6%)
Pediatrics, n (%)	15 (9.6%)	12 (11.8%)	3 (5.6%)	9 (7.3%)	7 (8.2%)	2 (5.3%)
MedPeds, n (%)	2 (1.3%)	0 (0%)	2 (3.7%)	0 (0%)	0 (0%)	0 (0%)
Emergency, n (%)	10 (6.4%)	10 (9.8%)	0 (0%)	8 (6.5%)	7 (8.2%)	1 (2.6%)
Physical medicine and rehabilitation, n (%)	17 (11%)	10 (9.8%)	7 (12.9%)	11 (8.9%)	10 (11.8%)	1 (2.6%)
Other, n (%)	6 (3.8%)	3 (2.9%)	3 (5.6%)	3 (2.4%)	2 (2.4%)	1 (2.6%)

Program evaluation 

The survey tool was approved as exempt by the Institutional Review Board. 

Participants were asked to complete a pre- and post-event survey (Appendix 1 and Appendix 2) to assess how well the virtual boot camp taught WM information, including their comfort in different WM topics. These topics were covered within the boot camp agenda and included altitude sickness, cold injury, heat injury, improvisational splinting, extraction decision-making, and mass casualty preparedness. Answers were assessed using a five-point Likert scale. An answer of five indicated the participant felt “very comfortable” with a specific topic and a score of one specified “not very comfortable.” Similar survey questions have been reported to evaluate previous fellowship boot camps [3,4]. Knowledge of these topics were also assessed using a pre- and post-boot camp test, completed simultaneously with the survey (Appendix 3). Two test questions were asked for each topic. 

The statistical program R version 4.1.1 (R Core Team, R Foundation for Statistical Computing, Vienna, Austria) was used along with a suite of tidyverse packages19 (primarily developed by Hadley Wickham; RStudio, Posit, Boston, MA) to perform data wrangling and produce a reproducible statistical analysis. Wilcoxon rank sum test, Fisher's exact test, and Pearson's chi-squared test were utilized to assess for statistical significance (p < 0.05). 

Because our competency ratings used ordinal Likert scale data and because normality could not be assured, we employed non-parametric Wilcoxon signed-rank tests (for paired comparisons pre-/post-intervention). For nominal categorical variables, we used Fisher’s exact test when counts were small (i.e., expected cell frequencies <5) and Pearson’s chi-squared test otherwise. These choices of statistical tests adhere to conventional guidelines for each type of data, thus ensuring appropriate handling of data distributions and sample sizes.

## Results

Of the 199 participants registered for the 2020 boot camp, 156 completed the pre-survey, and 129 completed the post-survey (78.4% and 64.8%, respectively). For the 2021 boot camp (131 total registered), 123 completed the pre-survey, and 118 completed the post-survey (93.9% and 90.0%, respectively). 

Statistical analysis was completed on participants who completed both the pre- and post-surveys. Those who did not complete either the pre- or post-survey were excluded from the analysis. This resulted in 110 complete surveys in 2020 (73 fellows and 37 attendings) and 91 complete surveys in 2021 (61 fellows and 30 attendings).

**Figure 1:**
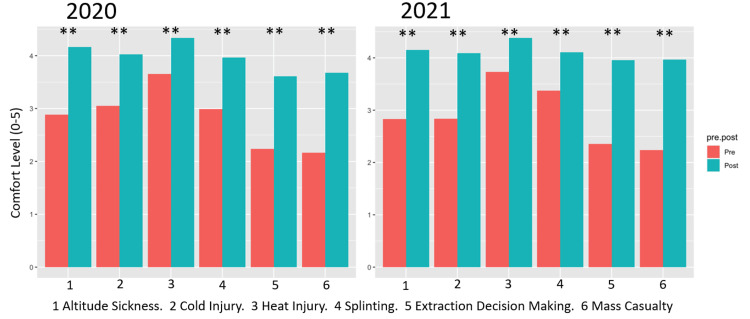
Fellow Comfort With Wilderness Medicine Topics, Pre- vs. Post-boot Camp by Year p-values of <0.001 are represented by **, and <0.05 represented by *.

**Figure 2:**
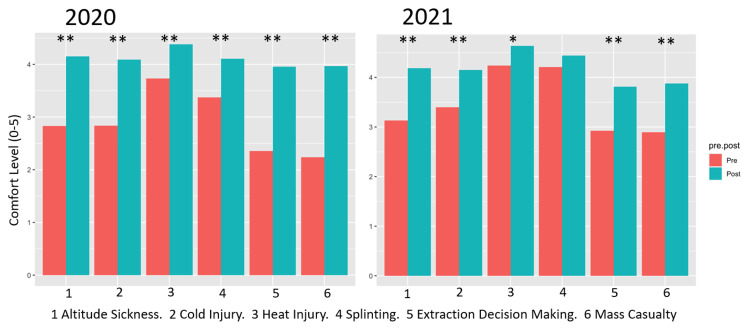
Faculty Comfort With Various Wilderness Medicine Topics, Pre- vs. Post-boot Camp by Year p-values of <0.001 are represented by **, and <0.05 represented by *.

Results of the survey revealed statistically significant (p < 0.05) improvement in comfort nearly universally, for fellows and attendings, for both years. The only result that was not statistically significant was the attendings’ rating of comfort improvement of improvisational splinting in 2021. Results of the comfort survey are presented in Table 4 and Figures 1-2. 

**Table 4:** Participant Comfort of Wilderness Medicine Topics Pre- and Post-boot Camp by Year Values in (parentheses) are standard deviations, followed by confidence intervals. Values in {brackets} are test statistics.

	2020						2021					
	Fellows			Faculty			Fellows			Faculty		
	Pre	Post	p-value	Pre	Post	p-value	Pre	Post	p-value	Pre	Post	p-value
Altitude sickness	2.85 (0.95; 2.63, 3.07)	4.19 (0.62;4.05, 4.34)	<0.001 {1830}	3.16 (0.79; 2.90, 3.42)	4.26 (0.64; 4.05, 4.47)	<0.001 {496}	2.84 (1.13; 2.55, 3.12)	4.21 (0.73; 4.03, 4.40)	<0.001 {1081}	3.10 (1.06; 2.70, 3.50)	4.27 (0.64; 4.03, 4.51)	<0.001 {300}
Cold injury	3.08 (0.85; 2.88, 3.28)	4.03 (0.55; 3.90, 4.1)	<0.001 {1485}	3.45 (0.86; 3.16, 3.73)	4.30 (0.66; 4.07, 4.50)	<0.001 {366}	2.82 (0.96; 2.57, 3.06)	4.13 (0.72; 3.95, 4.32)	<0.001 {1128}	3.40 (1.16; 2.97, 3.83)	4.17 (0.59; 3.95, 4.39)	<0.001 {226}
Heat injury	3.73 (0.90; 3.52, 3.94)	4.38 (0.54; 4.26, 4.51)	<0.001 {789}	4.18 (0.77; 3.93, 4.44)	4.61 (0.59; 4.41, 4.80)	<0.001 {105}	3.69 (0.96; 3.44, 3.93)	4.38 (0.64; 4.21, 4.54)	<0.001 {571}	4.27 (0.83; 3.96, 4.58)	4.63 (0.49; 4.45, 4.82)	0.009 {60}
Improvisational splinting	2.97 (1.00; 2.74, 3.21)	4.01 (0.77; 3.83, 4.19)	<0.001 {1362}	3.89 (0.86; 3.61, 4.18)	4.47 (0.65; 4.26, 4.69)	<0.001 {147}	3.44 (0.96; 3.20, 3.69)	4.16 (0.76; 3.97, 4.36)	<0.001 {812}	4.17 (0.99; 3.80, 4.53)	4.43 (0.82; 4.13, 4.74)	0.128 {58}
Extraction and decision-making	2.16 (0.85; 1.97, 2.36)	3.66 (0.89; 3.45, 3.86)	<0.001 {1953}	2.84 (0.95; 2.53, 3.15)	4.13 (0.88; 3.84, 4.42)	<0.001 {478}	2.31 (0.92; 2.08, 2.55)	4.08 (0.80; 3.88, 4.29)	<0.001 {1431}	3.00 (1.08; 2.60, 3.40)	3.87 (0.97; 3.50, 4.23)	<0.001 {208}
Mass casualty	2.16 (1.01; 1.93, 2.40)	3.75 (0.85; 3.56, 3.95)	<0.001 {2211}	2.68 (0.99, 2.36, 3.01)	3.92 (0.78; 3.66, 4.18)	<0.001 {594}	2.26 (1.12; 1.97, 2.55)	4.02 (0.76; 3.82, 4.21)	<0.001 {1586}	2.93 (1.20; 2.48, 3.38)	3.87 (0.82; 3.56, 4.17)	<0.001 {224}

**Figure 3:**
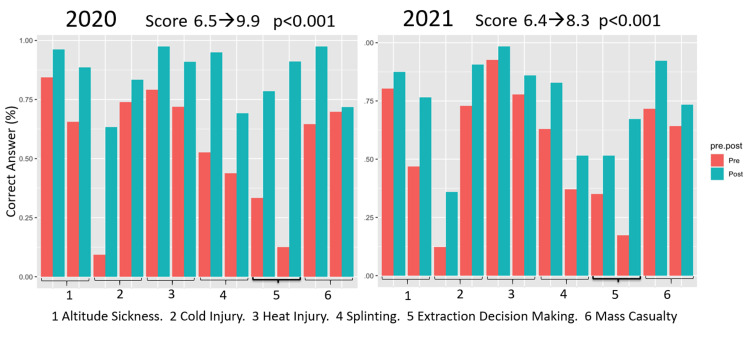
Fellow Knowledge With Various Wilderness Medicine Topics, Pre- vs. Post-boot Camp

**Figure 4:**
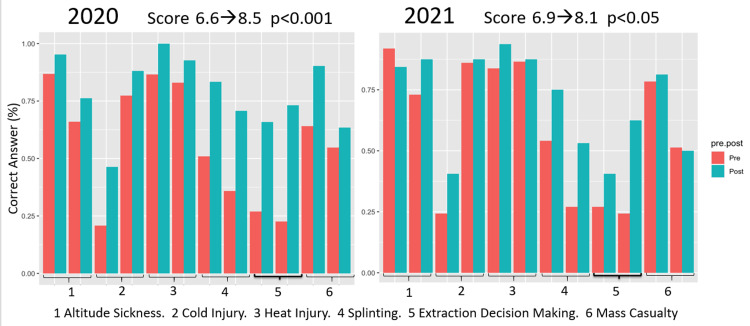
Faculty Knowledge With Various Wilderness Medicine Topics, Pre- vs. Post-boot Camp

The results of the knowledge test also showed statistically significant (p < 0.05) improvements following the boot camp (Figures 3-4). As there were 12 questions (two questions for each of the six previously listed topics), a score of 12 would indicate a perfect score. Fellows were seen to improve their test performance from 6.5 to 9.9 and 6.4 to 8.3 in 2020 and 2021, respectively (p < 0.001). Similarly, attendings improved their score from 6.6 to 8.5 in 2020 (p < 0.001) and 6.9 to 8.1 in 2021 (p < 0.05).

## Discussion

The COVID-19 pandemic created unprecedented challenges for teaching [8]. The need for social distancing and isolation prohibited traditional in-person learning and urged educators to adopt virtual teaching platforms [9]. Residency programs across an array of specialties similarly adopted online platforms. Early data during the pandemic from general surgery, neurosurgery, urology, and plastic surgery programs suggested that even surgical and hands-on skills learning can potentially be transitioned to the virtual sphere [10-14]. Our study demonstrated that a virtual WM boot camp can effectively teach WM concepts to a group of fellows and attendings and further supports that virtual learning can be used to learn and provide medical education. 

It has been demonstrated that in-person boot camps are an effective educational tool across various topics within graduate medical education [15-17]. Ours is among the first to show that virtual boot camps can similarly provide statistically significant results in medical education, specifically in the context of WM. The results of our study are based on two data sets - comfort with WM topics before and after attending the virtual boot camp and test scores in WM topics from before and after attending. Comfort level with the six assessed WM topics was nearly universally improved after boot camp attendance. It is unsurprising that the perceived level of understanding would be greater in the time immediately following an intensive educational experience on the topics in question. For fellows, comfort was improved in all areas (Figure 1). The one area in which comfort was not improved was improvisational splinting among attending physicians (Figure 2). The reason for this is likely multifactorial, due largely to the already high level of comfort that attendings had with this topic (score of 4.17 of five on preassessment), as well as the challenge associated with transferring this particularly hands-on skill into the virtual environment. Splinting is a dynamic, hands-on process that requires physical evaluation of particular pathology and body habitus, as well as the application of available equipment. The addition of more videos demonstrating splinting techniques may have allowed for a better understanding of improvisational splinting and further improved comfort post-boot camp. Other topics, including altitude sickness, cold injury, heat injury, decision-making, and mass casualty incidents, rely more heavily on medical decision-making and can be simulated in the form of clinical vignettes to discuss virtually.

Test scores from assessments conducted before and after the conference provided a more objective evaluation of learners’ understanding of WM topics. The data showed that across all learners, there was a statistically significant improvement in all six fields (Figures 3 and 4). This is an encouraging finding, demonstrating that medical education can be effectively delivered via online education. It supports the continued development and implementation of virtual learning for graduate medical education. The success of the virtual WM boot camp underscores the adaptability and efficacy of virtual learning in a field that traditionally relies on hands-on experiences. This transition not only facilitated the continuation of essential medical education but also provided insights into the broader implications and possibilities of virtual medical education in a post-pandemic world. 

The study population is generalizable to PCSM fellowships as it involved a diverse range of fellows across the USA. Fellowships of differing sizes, different time zones, and geographic regions were able to participate. As this was a free event, it was the author's hope that this event was able to be equitable for all programs, even those who may identify as under-resourced. As the only need for participation was a device with internet access, it was believed that there would be no access limitations to any program within the USA. 

Implications for wilderness medicine education

Online learning offers a solution to significant hurdles posed by traditional WM education. There are significant cost savings associated with virtual learning [18]. Participating online eliminates the financial burden imposed by traveling to remote learning environments, often including airfare, accommodation, and other incidental expenses. Other savings might include fees associated with ski resorts, parks, private land, and other fee-restricted areas. 

As it pertains specifically to PCSM fellowships, WM teaching and experience are limited for many programs. Due to the significant expense of travel and resources, as well as time away from the busy fellowship schedule, programs may not be able to offer WM teaching to their fellows. By creating the virtual event, we were able to provide a robust WM experience to a wide range of fellows and eliminate the constraints that previously made WM experiences unobtainable. Unfortunately, no data is available regarding the cost of away rotations for fellows to achieve WM experiences. However, several studies have been performed on away rotations for medical students and residents [19-21]. While total costs varied widely, their reported findings ranged from an average of $958 to $3591. When travel, lodging, food, and other expenses are considered, the cost of a WM rotation is likely within that range. Additionally, this cost cannot include the incalculable expense of time away from clinical responsibilities and family. 

The online platform also frees up valuable time for participants and educators to engage in education. Travel alone can be a significant hurdle for individuals participating in traditional WM experiences. Furthermore, in-person training must factor in logistical burdens that may be imposed by travel delays, inclement weather, and other unforeseen circumstances, much of which are eliminated in the controlled environment of the virtual learning space. Our virtual boot camp ensured that participants received a scripted and intentionally comprehensive learning experience that would not have been affected by the aforementioned unforeseen circumstances. While connectivity to virtual platforms may be a challenge in some situations, no delays or complications related to internet connection were reported during the conference. As the boot camp took place in the USA, this was a fortunate ability that may not be feasible in other areas of the world. A recording of each presentation could be performed, so videos could be distributed at a later time if a connectivity issue occurs. 

As noted in the prior publication by Loeffert et al. [5], the virtual education platform allows for the recruitment and inclusion of a much broader audience. Prior in-person boot camps published survey results of relatively small sample sizes (i.e., six to 47 participants). To the authors’ knowledge, this boot camp remains the largest studied boot camp to date. The scalability of virtual education is exponential, limited only by the platform used for audience attendance that may have a maximum attendee limit. Other areas of medical education may be able to reach a broader and larger attendance size by using a virtual platform.

Finally, the virtual format is inclusive. It facilitates participation from a larger, more diverse, and more geographically dispersed audience, providing more individuals with essential WM education than in-person experiences might. The flexibility of virtual learning allows participants to engage with the material from the comfort of their desired learning location, streamlining the learning process and accommodating varied schedules. We were also able to overcome challenges for participants located in different time zones by offering an intentionally later start time so those located Pacific time could realistically participate. The inclusivity and adaptability inherent in virtual WM education not only optimize logistical aspects but also foster a more accessible and widespread dissemination of essential medical knowledge in austere environments. It was not needed for our event, but recording presentations to be watched later could be an option if participants are too geographically separated to make watching a live presentation unrealistic. 

Limitations and future directions 

Despite the benefits discussed, our virtual WM boot camp format is not without limitations. One drawback is that participants were unable to interact with and manipulate equipment physically, missing out on the opportunity to familiarize themselves with tools and techniques that may be encountered in real-world scenarios, like improvisational splinting. While presenters made efforts to convey these experiences through visual aids and descriptive language, the virtual platform struggles to emulate the tangible, in-the-field interactions found in traditional settings. Additionally, the virtual environment lacks the practicality of simulated scenarios carried out in real-time. Traditionally, in these in-person simulations, participants collaboratively address and work through simulated WM cases, recreating the team-based care and real-time urgency encountered in real-life practice. 

Our study primarily focused on immediate changes in participants' comfort and knowledge of WM topics following the boot camp. The long-term retention and application of this knowledge in clinical practice remain uncertain. An ongoing investigation should incorporate follow-up surveys to investigate how participants utilize the knowledge they have gained to enhance their clinical abilities or integrate their experiences into practice six or 12 months post-boot camp. It would be of value to create a knowledge test describing real-world clinical scenarios and addressing how participants score six or 12 months post-boot camp. Additionally, as our study evaluated survey results, it cannot be said to be without bias. Given the limitations of self-reported data and the absence of long-term follow-up, the findings should be interpreted as demonstrating the immediate potential of virtual education rather than definitive evidence of its equivalence to in-person training. Future studies should incorporate direct comparisons, long-term assessments, and objective measures to validate these results further.

Our study evaluated a single boot camp that was performed two years in a row at a single institution. For this reason, the results may not be generalizable to all medical education events. The quality of the boot camp met high standards set by governing bodies that oversee and grant Continuing Medical Education (CME) certification. It is our hope that by achieving CME requirements and including presenters with experience providing virtual education presentations, similar outcomes can be achieved. 

It should be noted that a person's ability to participate in a virtual program requires the ability to have a device compatible with video software and internet access. Fortunately, this did not seem to be a challenge for our target population. However, it could present challenges in areas with limited internet access or technical resources, which could impact the replicability of the intervention globally.

In addition to the limitations discussed, the reliance on self-reported data introduces a potential bias, as participants may overestimate their comfort and knowledge. Future studies could incorporate objective performance evaluations to complement survey findings. Additionally, while our study demonstrates the feasibility of virtual education within the USA, technological disparities in resource-limited settings may pose barriers to replicability on a global scale. Addressing these challenges through enhanced technical support and asynchronous learning options should be explored in future iterations of the program.

Finally, while this research provides valuable insights into the effectiveness of virtual education in WM, it is important to note that we did not directly compare the effectiveness of virtual to in-person education modalities. Future studies should compare the effectiveness of virtual learning to that of in-person experiences. It remains unclear if these modes of teaching provide similarities in education outcomes. These studies could include either randomized control or cross-over design studies. 

Our findings suggest that virtual education is an effective tool for immediate knowledge and comfort improvement in WM. However, further research is needed to evaluate the retention of knowledge and its application in clinical practice. While our study supports the feasibility and benefits of virtual WM education, the efficacy of virtual methods compared to in-person training remains to be established. Although the improvements were statistically significant, the moderate effect sizes suggest that virtual education serves as a meaningful supplement to, but not a full replacement for, hands-on training in certain areas like improvisational splinting.

As technology continues to advance and the landscape of medical education evolves, ongoing research efforts should aim to provide a holistic understanding of the strengths and limitations of virtual education in various medical specialties. This will not only contribute to evidence-based educational practices but also guide the development of future virtual education initiatives.

## Conclusions

We were able to demonstrate that our virtual WM curriculum effectively increased comfort with and knowledge of WM topics for those who participated immediately following the boot camp. The findings strongly support our hypothesis that virtual platforms are an efficacious and viable tool for education. While it remains uncertain how virtual education compares to in-person boot camps, this study supports the possibility that virtual education may be an acceptable alternative in certain situations. These platforms offer numerous advantages, though future research should focus on comparing the effectiveness of virtual versus in-person education modalities to inform evidence-based educational practices. By advancing our understanding of virtual education implementation, we can ensure that learners more broadly receive comprehensive and effective education regardless of the learning environment.

## Appendices

Appendix 1 

**Table 5:** Pre-Event Survey

	Question	Response options
1	What are the last four digits of your cell number? (Please remember what number you enter - you will be asked to use it again in the post-training survey.)	Open text
2	What is your age?	Open text
3	What is your gender?	Male. Female. Non-conforming.
4	Please select your medical specialty.	Family medicine. Internal medicine. Pediatrics. MedPeds. Emergency. Physical medicine. Rehabilitation. Others.
5	You are a(n):	Fellow. Attending. Resident.
6	How are you attending this boot camp?	In-person. Virtually.
7	Number of years since fellowship graduation OR since residency if no fellowship.	Open text
8	State where you grew up.	Open text
9	State where you lived the longest.	Open text
10	Do you have any prior experience with Boy/Girl Scouts (or other outdoor-focused organizations)?	Yes. No.
11	Do you have any previous education in wilderness medicine?	Yes. No.
12	Do you enjoy outdoor activities?	Yes. No.
13	Please identify your current level of comfort for each of the following concepts. (1 - not very comfortable to 5 - very comfortable)	Altitude sickness. Cold injury. Heat injury. Splinting. Extraction and decision-making. Mass casualty.

Appendix 2 

**Table 6:** Post-Event Survey

	Question	Response options
1	What are the last four digits of your cell number? (This should be the same number you used for the pre-training survey.)	Open text
2	Did you complete the pre-boot camp survey?	Yes, No
3	What is your age?	Open text
4	What is your gender?	Male. Female. Non-conforming.
5	Please select your medical specialty.	Family medicine. Internal medicine. Pediatrics. MedPeds. Emergency. Physical medicine. Rehabilitation. Others.
6	You are a(n):	Fellow. Attending. Resident.
7	Number of years since fellowship graduation OR since residency if no fellowship.	Open text
8	State where you grew up.	Open text
9	State where you lived the longest.	Open text
10	Do you have any prior experience with Boy/Girls Scouts (or other outdoor-focused organizations)?	Yes. No.
11	Do you have any previous education in wilderness medicine?	Yes. No.
12	Do you enjoy outdoor activities?	Yes. No.
13	Please identify your current level of comfort for each of the following concepts. (1 - not very comfortable to 5 - very comfortable)	Altitude sickness. Cold injury. Heat injury. Splinting. Extraction and decision-making. Mass casualty.
14	Please answer the following questions about the boot camp: (1 - strongly disagree to 5 - strongly agree)	I feel this boot camp was useful in developing my knowledge of wilderness medicine. I feel this boot camp was useful in developing my self-confidence. I feel this boot camp improved my ability to care for patients in different environments. I believe this information is important for primary care sports medicine fellows. I would recommend this boot camp to future fellows. I feel today’s boot camp adequately taught wilderness medicine topics. I feel the virtual experience was sufficient to learn wilderness medicine topics (i.e., does not need to be done in person).
15	How are wilderness medicine topics taught in your program? (Check all that apply.)	A formal wilderness medicine fellowship program. Didactic sessions. Journal club. Clinical teaching sessions. Away rotations. Others, please specify. We do not cover these topics; please specify which are not currently covered in your program.
16	I would be interested in having our fellowship attend an in-person wilderness medicine boot camp near Hershey, PA, next year.	Yes - Great! We thank you for your interest. Please email Dr. Loeffert in order to in join the list for information about next year. jloeffert@pennstatehealth.psu.edu. No.
17	I would be interested in our program attending a virtual wilderness medicine boot camp next year.	Yes. No.
18	To improve this event in the future, I recommend:	Open text

Appendix 3 

**Table 7:** Pre-/Post-Boot Camp Knowledge Test Questions

	Question	Response options
1	What is the first-line treatment for all altitude-borne illnesses?	Dexamethasone. Descent. Acetazolamide. Acclimatization.
2	Although data are limited, which of the following environments have been shown to improve athletic performance?	Live low, train low. Live low, train high. Live high, train high. Live high, train low.
3	You have just been assigned to a crew of field searchers. The crew is composed of two to four skilled searchers who have had a great deal of training and are capable of moving very quickly over the terrain to which you will be assigned. Your task is to search a travel route the subject may have used. Your crew is said to be on:	Loose grid search. Tight grid search. Hasty search. Trial patrol.
4	Which of the following carry techniques require more than one rescuer?	Blanket drag. Fireman’s carry. Web sling. Fore-and-aft carry.
5	Which of the following is not a part of the Simple Triage and Rapid Treatment (START) algorithm?	Red: immediate - acute life-threatening injury. Yellow: delayed - injured, less critical can wait. Green: minimal - mild injury - walking wounded. Blue: no injury - completely well. Black: expectant/deceased.
6	Which is the most common cause of injury in a mass casualty incident?	Head injury. Hemorrhage. Cardiac event. Pneumothorax.
7	What is the most important mechanism to lose heat?	Evaporation. Radiation. Conduction. Convection.
8	A person suffering from heat stroke should have their temperature dropped to <40ºC in what amount of time?	15 minutes. 30 minutes. 45 minutes. 60 minutes.
9	In a patient who has frostbite in the field, with definitive care more than two hours away and a low likelihood of refreezing, the best course of action is:	Rapid rewarming via warm water immersion with water maintained at a temperature of 37-39ºC. Rapid rewarming via warm water immersion with water maintained at a temperature of 40-42ºC. Slow rewarming via lukewarm water immersion. Rapid rewarming via radiation using a heat source such as fire, heater, etc. Slow rewarming using body heat and friction, including gently rubbing the affected areas.
10	Which of the following is a contraindication to cardiopulmonary resuscitation in a severely hypothermic patient?	Fixed-dilated pupils. Apparent rigor mortis. Snow burial >35 minutes. Dependent lividity.
11	In the event of a snake bite, the following is the recommended initial field management:	With an available clean-cutting tool, make an incision across the puncture marks and attempt to oral extract the venom. With a mechanical suction device, attempt to extract whatever venom you can. Keep the patient calm, call poison control, and begin evacuating to the nearest medical facility. To facilitate definitive care and administration of anti-venom, kill the snake so proper identification can be made. Apply a tourniquet to limit the systemic spread of the venom.
12	If caught in the field during a thunderstorm, your best course of action is to:	Assume the "lightening position." This involves sitting or crouching with feet and knees together and insulating oneself from the ground by sitting on a pack or foam pad. Seek shelter in the nearest building away from doors and windows. Stand next to a large tree to minimize your contact with the ground while being lower than the tree. If in a group, huddle together close to the ground to minimize a lightning rod effect for each individual. There is no safe place from lightning, so it does not matter. Just hope for the best.
